# A Bio-Inspired Weather-System Sensing Framework for Physically Constrained Precipitation Nowcasting Correction

**DOI:** 10.3390/biomimetics11080526

**Published:** 2026-07-24

**Authors:** Youming Qu, Xian Feng, Linyan Luo, Xun Deng, Runqing Kang, Guanru Lv, Jiachi Shi, Wei Peng, Jianhong Gan, Kun Cai, Peiyang Wei, Zhibin Li

**Affiliations:** 1 Changde Meteorological Bureau, Changde 415000, China; 2 Hunan Meteorological Data Center, Hunan Meteorological Bureau, Changsha 410118, China; 3 University of Chinese Academy of Sciences, Beijing 100049, China; 4 College of Software Engineering, Chengdu University of Information Technology, Chengdu 610225, China; 5 Sichuan Key Laboratory of Software Automatic Generation and Intelligent Service, Chengdu University of Information Technology, Chengdu 610225, China; 6 Key Laboratory of Meteorological Software China Meteorological Administration, Chengdu 610225, China; 7 Dazhou Key Laboratory of Government Data Security, Sichuan University of Arts and Science, Dazhou 635000, China

**Keywords:** bio-inspired intelligence, weather-system sensing, precipitation nowcasting correction, physical constraints, jet-stream forcing, interpretable deep learning

## Abstract

Accurate correction of gridded numerical weather prediction precipitation forecasts remains challenging because many end-to-end deep learning correction models treat meteorological variables as undifferentiated data channels and therefore provide limited physical interpretability. Inspired by general principles of biological environmental sensing, selective information processing, and regulatory constraint learning, this study proposes PCPNet, a bio-inspired and physically constrained precipitation correction framework. The framework does not imitate a specific biological organ or species; instead, it abstracts three information-processing principles into a meteorological correction task. First, key weather-system cues, including low-level shear lines, trough-ridge effects, upper-level jet-stream forcing, vorticity-divergence-related vertical motion, and water-vapor flux convergence, are quantified as structured diagnostic fields. This transforms the subjective synoptic diagnosis of forecasters into automated grid-based sensing features. Second, these diagnostic cues are fused with numerical weather prediction variables and terrain descriptors in an encoder–attention–decoder network, allowing the model to emphasize dynamically important precipitation-triggering regions. Third, water-vapor conservation and terrain-forcing relationships are embedded as differentiable constraint losses, providing training-time constraint-based regulation that guides the corrected precipitation field toward physically consistent solutions. The method is evaluated from 2021 to 2023 in Hunan Province, China, using hourly numerical weather prediction model outputs as input features, China Meteorological Administration Land Data Assimilation System gridded analysis data as the training target, and independent meteorological station observations for strict cross-validation. PCPNet reduces the mean absolute error by 22.1% compared with the uncorrected China Meteorological Administration Land Data Assimilation System gridded precipitation products and outperforms Linear Regression, Bagging, Boosting, Multi-Layer Perceptron, TabNet, and Tree-based Progressive Regression Models by 12.9%, 13.5%, 16.9%, 10.8%, 14.9%, and 15.9%, respectively. The single-day event analysis provides an initial demonstration of heavy precipitation recovery capability, while comprehensive validation across long-term continuous weather events is planned for future operational deployment to further verify model stability. These results indicate that bio-inspired sensing and regulatory constraint learning can improve both the accuracy and interpretability of precipitation nowcasting correction.

## 1. Introduction

Precipitation is one of the core variables in meteorological forecasting, climate analysis, and hydrological applications. Numerical weather prediction (NWP) models serve as the central tools in modern meteorological operations, and the grid-based precipitation forecasts they output form the foundation for various applications. However, due to factors such as initial condition errors, parameterization simplifications, and complex terrain, the raw precipitation forecasts from these models often exhibit significant biases. These biases manifest in the following ways: inaccurate capture of the dynamic triggering mechanisms of precipitation systems (such as shear lines and upper-level jet streams), resulting in displaced precipitation areas; errors in the quantitative description of water vapor transport and convergence, leading to misrepresented intensity; and insufficient representation of mesoscale terrain lifting effects, causing unrealistic precipitation distributions on the windward and leeward slopes.

Bio-inspired systems provide a useful conceptual perspective for dealing with complex, uncertain, and dynamically changing environments. Rather than relying on a single cue, living systems often integrate heterogeneous environmental signals, selectively emphasize salient information, and maintain stable responses through regulatory constraints. Biomimetics and bio-inspired engineering transfer such principles from biological systems into technical designs [1], and attention-inspired models have also shown how selective processing can reduce complex scene analysis to salient regions [2]. In precipitation correction, a similar abstraction can be introduced: weather-system diagnostics can be regarded as environmental sensing cues, attention-based feature fusion as selective information processing, and physical constraint losses as training-time regulation that discourages unrealistic outputs.

The objective post-processing of numerical weather prediction output is a critical procedure in meteorology to mitigate systematic model biases and address the uncertainty inherent in atmospheric parameterization schemes. In practice, due to the limitations of observational instruments, the diversity of observation environments, and errors in data processing, raw meteorological data often contain biases, making it difficult to use them directly for high-precision weather forecasting and climate research. To improve the accuracy and reliability of meteorological data, meteorologists employ real-time data correction techniques, which involve a series of scientific procedures such as data quality control, error analysis, and data assimilation to eliminate or minimize errors. This work is of significant importance for enhancing weather forecast accuracy and advancing meteorological research. As technology advances and meteorological observation methods diversify, the correction of real-time meteorological data faces new challenges and opportunities, requiring continuous innovation and improvement.

Currently, there is relatively limited research on the correction of grid-based meteorological real-time data. This study specifically focuses on the bias correction of grid-based numerical weather prediction forecasts, utilizing historical model outputs generated with a fixed lead time and corresponding high-resolution gridded analysis fields to establish a robust mapping relationship. Common grid-based forecast correction techniques include Model Output Statistics (MOS) [3,4,5], Kalman filtering [6], neural networks [7], site-to-grid correction methods, optimal ensemble forecast correction, the East China mean method, spatial attribute-based correction techniques, and correction methods based on machine learning [8]. For example, Huang et al. proposed the Bernoulli–Gamma–Gaussian model [9] to assess the applicability of the next-generation GFDL-SPEAR model in the secondary water resource areas of China. The corrected GFDL-SPEAR precipitation forecast provided key decision support for watershed water resource management and flood and drought prevention, extending the forecast lead time from six months to one year. In recent years, bias correction has become an important research field in meteorology, especially in the context of using reanalysis data (such as ERA5) for model output calibration and validation. With the advancement of the Coupled Model Intercomparison Project Phase 6 (CMIP6), many studies combine CMIP6 data with reanalysis data to assess model biases in different regions and climate variables, further improving forecast accuracy through bias correction techniques [10]. However, traditional statistical methods (such as the Bernoulli–Gamma–Gaussian model) often rely on prior assumptions about data distributions, which can be limiting when applied to meteorological data. The true distributions of meteorological data are often highly nonlinear and random, leading to challenges in stability, generalization, timeliness, and accuracy when using traditional methods [11]. Therefore, existing statistical methods face difficulties in handling complex meteorological data, and there is an urgent need for more flexible and efficient correction techniques.

With the rapid development of deep learning technology, models such as Convolutional Neural Networks (CNN) [12], Recurrent Neural Networks (RNN) [13], Deep Neural Networks (DNN) [14], and Transformers have provided new data-driven methods for correcting precipitation data. Deep learning models possess powerful nonlinear modeling capabilities, enabling them to automatically learn complex features and mapping relationships from large-scale observational data [7]. As a result, they have shown great potential in the field of meteorological data correction. For example, Tan et al. proposed a deep learning model called NFC-Net [15] for correcting the numerical weather prediction results from the European Centre for Medium-Range Weather Forecasts (ECMWF), significantly improving forecast accuracy. Similarly, Zhao et al. applied the Bernoulli–Gamma–Gaussian model to correct ECMWF precipitation forecast errors [16], further improving forecast accuracy. Most deep learning methods used for meteorological data correction are end-to-end “black-box” models. These models cannot explicitly represent key meteorological systems and therefore offer limited meteorological interpretability, which can affect their accuracy. The spatiotemporal organization of short-duration heavy rainfall is also strongly modulated by terrain [17], reinforcing the need to incorporate physically meaningful weather-system and topographic cues into correction models. Recent advancements in operational meteorological modeling emphasize the necessity of physically constrained frameworks, such as high-performance multi-scale coupling architectures for high-fidelity real-time simulations [18]. Furthermore, understanding broader environmental interactions, including biophysical feedbacks on precipitation patterns [19], continues to drive the refinement of grid-based correction techniques.

To address the aforementioned issues, this paper proposes PCPNet, a bio-inspired weather-system sensing and physically constrained precipitation correction framework. The core idea is to abstract three principles from adaptive living systems—multi-cue environmental sensing, selective information processing, and regulatory feedback—and transfer them into grid-based precipitation correction. The proposed method is not a direct imitation of a specific organism; rather, it uses bio-inspired information-processing principles to organize meteorological diagnostic cues, attention-based feature fusion, and physical constraint learning. The main innovations and contributions of this paper are as follows:

(1) This paper introduces a bio-inspired sensing–regulation–correction framework for precipitation nowcasting correction. By calculating specific dynamic and thermodynamic proxies, including low-level relative vorticity and upper-level wind speed indices, key atmospheric structural patterns are mapped into grid-based objective variables to assist model learning.
(2) This paper constructs a unified multi-dimensional feature tensor and designs an end-to-end encoder–attention–decoder model to integrate multi-source meteorological variables, terrain descriptors, and diagnostic sensing cues. The attention module allows the model to focus on dynamically salient precipitation-triggering regions, analogous to selective attention in biological perception.
(3) This paper integrates core physical laws, such as water vapor conservation and terrain forcing, into the model training process as differentiable loss functions. These constraints act as training-time regulators that guide the corrected precipitation field toward physically plausible solutions while minimizing numerical errors.

## 2. Related Work

### 2.1. Multi-Layer Perceptron

The Multi-Layer Perceptron (MLP) is a common artificial neural network model consisting of multiple layers of neurons. It is a feedforward neural network, where information flows unidirectionally from the input layer through hidden layers to the output layer [20]. Each layer contains multiple neurons, and the hidden and output layers typically employ nonlinear activation functions. The training process of MLP usually utilizes the backpropagation algorithm, which adjusts the weights and biases in the network by minimizing the loss function to reduce the difference between the model’s predictions and the true labels. MLP has powerful nonlinear modeling capabilities, enabling it to approximate complex nonlinear relationships. However, MLP also faces several challenges, such as a tendency to overfit, the need for large amounts of training data, and the requirement to fine-tune numerous hyperparameters. Therefore, in practical applications, MLP is often enhanced and optimized by integrating other techniques and methods, depending on the specific application scenario.

### 2.2. Convolutional Neural Network

A Convolutional Neural Network (CNN) typically consists of basic components such as the input layer, convolutional layers, pooling layers, fully connected layers, and the output layer. In the convolutional layers, convolution kernels serve as weight matrices (e.g., in two-dimensional space, they are typically 3 × 3 matrices) [21]. These kernels slide over the original input matrix in a fixed manner to produce a new matrix. The convolutional layers use convolution operations combined with activation functions (such as Sigmoid or ReLU) to extract features [22]. At lower layers, CNNs extract low-level features, while higher layers generate more complex abstract features through convolutions of the low-level features. After the convolutional layers, pooling layers are added to enhance the model’s generalization ability. Pooling operations (such as max pooling or other techniques) reduce the resolution of the feature maps, ensuring the translational invariance of the CNN [23]. After several convolution and pooling layers, the data flows into fully connected layers, where high-dimensional abstract features are aggregated and the final results are output.

## 3. Method

**Figure 1:**
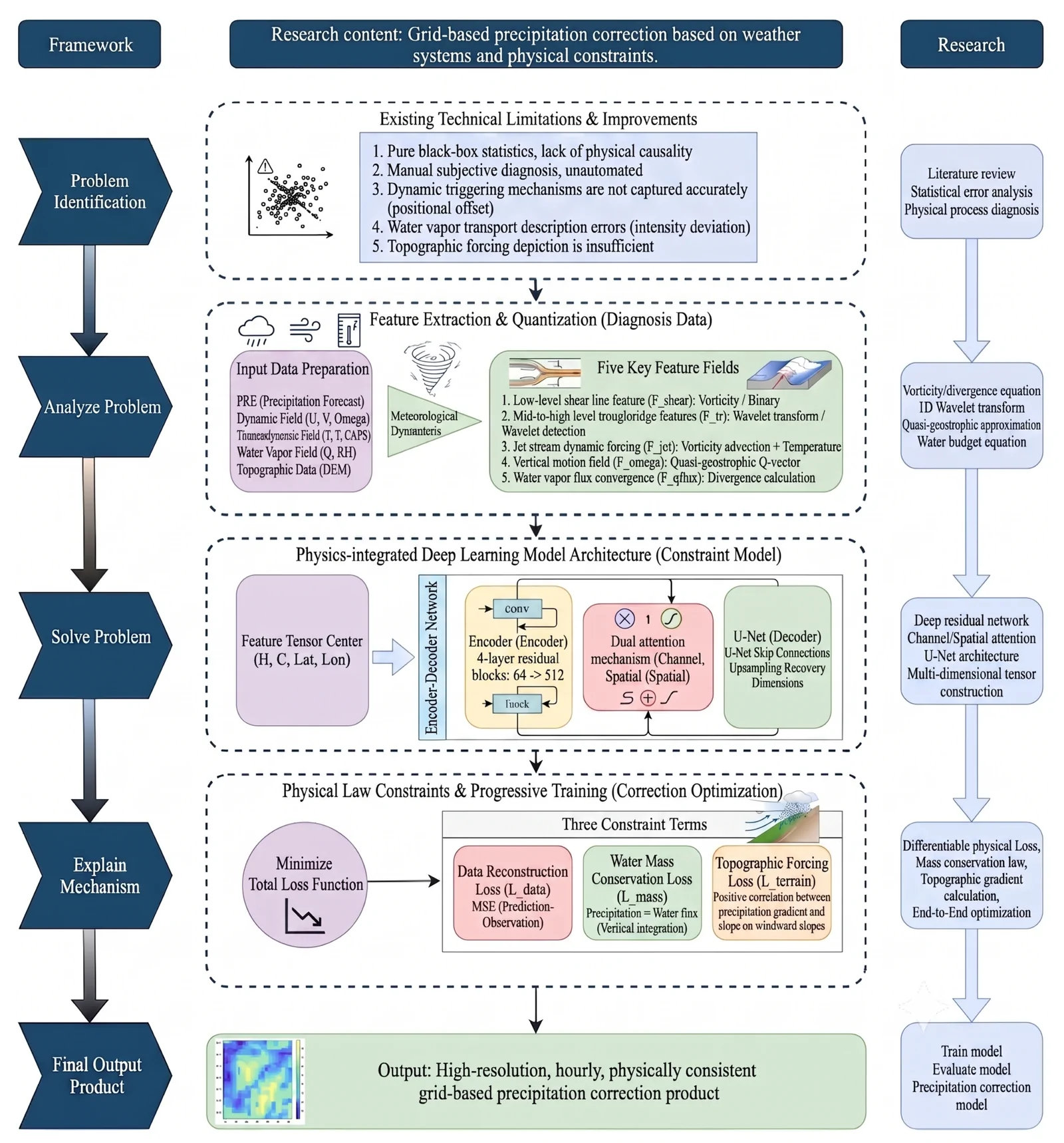
Overall technical roadmap. Arrows indicate the data and processing flow, while colored blocks distinguish the functional modules of the proposed framework.

This paper proposes a grid-based precipitation correction method that integrates weather system diagnostics with physical constraints, aiming to address the lack of physical interpretability in statistical correction methods and the difficulty of automating manual diagnostics. The method begins by extracting key weather system feature fields such as shear lines, troughs, ridges, jet stream forcing, vertical motion, and water vapor convergence from the meteorological fields output by the numerical model. This transforms subjective dynamic diagnostics into quantifiable grid features. These features are then stacked with the original meteorological fields and geographic information data to form a multi-dimensional tensor, which is input into an encoder–attention–decoder deep learning model. During the model optimization process, alongside the conventional data reconstruction error, simplified physical penalty terms representing primary terrain gradient forcing and column-integrated moisture convergence proxies are implemented. These physical laws are deeply embedded in the model optimization through differentiable loss functions. A technical roadmap of the method is shown in Figure 1.

### 3.1. Bio-Inspired Design Rationale

The proposed framework conceptualizes precipitation correction through an information-processing perspective, differentiating itself from standard physics-informed deep learning by deeply coupling automated synoptic diagnostics with constrained optimization. Rather than relying solely on data-driven attention weights to discover relevant features, the framework explicitly hardcodes meteorological priors into the input space via structured diagnostic fields such as low-level shear and jet-stream forcing. The added value of this approach lies in integrating these physically meaningful perception cues with differentiable physical losses. The attention module acts as a selective filter to emphasize dynamically salient regions [2], while the physical constraints serve as a regulatory mechanism during training to push the data-driven outputs toward physically plausible mass conservation and terrain-lifting states. The surrounding model structure follows the same cautious abstraction. Multi-cue environmental sensing motivates the construction of diagnostic weather-system fields from dynamic, thermal, moisture, and terrain-related variables. Constraint-based regulation motivates the use of water-vapor conservation and terrain-forcing terms as differentiable losses during training. These regulatory terms are not an inference-time closed feedback loop; they are training-time constraints that improve the physical plausibility of the learned correction function.

### 3.2. Data Preparation

The raw grid forecast products output by the numerical weather prediction model for the Hunan Province region are collected. The required base data include meteorological element fields and geographic information data. The meteorological element fields encompass: precipitation forecast field (PRE), geopotential height field (Z), horizontal wind field (U, V), specific humidity field (Q), relative humidity field (RH), vertical velocity field (OMEGA), convective available potential energy field (CAPE), surface pressure field (PSFC), and temperature field (T). The geographic information data include high-resolution digital elevation models (DEMs), slope, and aspect.

The input data undergo multi-step preprocessing, including standardizing the unified data format, geographic domain clipping, spatial interpolation, and metric coordinate transformation. First, all datasets are clipped to the research domain bounded by 24° N–31° N and 107° E–115° E. Second, bilinear interpolation is applied to unify all meteorological and geographic fields onto a regular 0.01° × 0.01° latitude–longitude grid.

Notably, raw geographic derivatives calculated directly on spherical latitude–longitude coordinates suffer nonuniform spatial metric spacing: the physical distance corresponding to 0.01° longitude shrinks with increasing latitude, which breaks the physical consistency of spatial gradient, vorticity, and divergence diagnostic calculations. To resolve this issue, we convert the spherical lat/lon grid to a local planar Cartesian metric coordinate system (units: meters) prior to computing all dynamic diagnostic feature fields.

For each grid point (λ,φ) (longitude λ, latitude φ), the zonal (x) and meridional (y) metric increments are calculated using a spherical-Earth approximation, as shown in Equation (1):

(1) $$\left\{\begin{array}{c} dx=R\cos\varphi \cdot \Delta \lambda \cdot \pi /180\\ dy=R\cdot \Delta \varphi \cdot \pi /180 \end{array}\right.$$

where R=6,371,000m denotes the mean Earth radius, $\Delta \lambda =0.01^{\circ },\Delta \varphi =0.01^{\circ }$.

### 3.3. Extraction and Quantification of Key Weather System Feature Fields

The background fields of weather systems play a crucial role in distinguishing this method from purely statistical approaches [24]. By performing systematic meteorological diagnostic calculations on the raw dynamic, thermal, and moisture fields, a series of intermediate feature fields with clear physical meaning are generated, which directly represent the dynamic, thermal, and moisture conditions influencing precipitation. The definitions and calculation methods for each feature field are as follows:

#### 3.3.1. Shear Line Feature Field ($F_{shear}$)

Based on the 850 hPa horizontal wind components (U, V), the relative vorticity $\zeta_{s}$ is computed as a simplified single-variable indicator of the general rotational intensity in the lower troposphere [25]. The formula is shown in Equation (2):

(2) $$\zeta_{s}=\frac{\partial v}{\partial x}-\frac{\partial u}{\partial y}$$

In Equation (2), *u* is the zonal wind component (units: m/s); *v* is the meridional wind component (units: m/s); and *x* and *y* represent the zonal and meridional Cartesian coordinates in the metric projection (units: m). Subsequently, a predefined vorticity threshold is used to binarize significant shear areas, and morphological closing operations are applied to connect adjacent regions, forming a continuous shear line spatial distribution. The resulting low-level shear line feature field ($F_{shear}$) quantitatively represents the intensity of low-level convergence, with high-value areas corresponding to potential uplift trigger zones.

#### 3.3.2. Mid-to-Upper-Level Trough-Ridge Impact Feature Field ($F_{tr}$)

For the 500 hPa geopotential height field (Z), a wavelet transform-based wave packet detection algorithm is used to identify relative low-value areas (troughs) and high-value areas (ridges) in the waves [26]. The trough area index is defined as +1, and the ridge area index is defined as −1. A Gaussian smoothing operation is applied to generate a spatially continuous trough-ridge impact index field $F_{tr}$. This field reflects the dynamic guidance and suppression effects of mid-to-upper atmospheric waves on precipitation systems.

#### 3.3.3. Upper-Level Jet Stream Dynamic Forcing Feature Field ($F_{jet}$)

For the 200 hPa upper-level wind field, regions where the wind speed exceeds 30 m/s are identified as jet axes. Special focus is placed on diagnosing secondary circulation forcing to the right of the jet entrance and to the left of the jet exit [27]. By coupling vorticity advection and temperature advection terms, the jet stream dynamic forcing feature field $F_{jet}$ is constructed. The formula is shown in Equation (3):

(3) $$F_{jet}=-V\cdot \nabla \left(\zeta +f\right)+\kappa \left(-V\cdot \nabla T\right)$$

In Equation (3), *V* is the horizontal wind vector (units: m/s); ∇ is the two-dimensional gradient operator; ζ is the relative vorticity (units: s^−1^); *f* is the Coriolis parameter (units: s^−1^); *T* is the temperature (units: *K*); and κ is a dimensionless thermal adjustment coefficient. High-value regions of $F_{jet}$ indicate strong upper-level divergence and corresponding suction effects.

#### 3.3.4. Quasi-Geostrophic Omega Equation Vertical Motion Field ($F_{omega}$)

To comprehensively diagnose the large-scale vertical motion potential, we adopt the classical Quasi-Geostrophic (QG) Omega Equation [28], formulated in pressure coordinates (*p*). This formulation integrates both dynamic forcing (differential geostrophic absolute vorticity advection) and thermodynamic forcing (the horizontal Laplacian of geostrophic thermal advection), regulated by the static stability parameter, thereby overcoming the limitations of single-level vorticity advection diagnostics. The diagnostic formulation for the vertical motion forcing field $F_{\omega }$ is defined in Equation (4).

(4) $$F_{\omega }=\frac{f_{0}}{\sigma }\frac{\partial }{\partial p}\left[V_{g}\cdot \nabla \left(\zeta_{g}+f\right)\right]+\frac{R}{\sigma p}\nabla^{2}\left[V_{g}\cdot \nabla T\right]$$

In Equation (4), $F_{\omega }$ represents the complete quasi-geostrophic forcing for vertical velocity (ω≡dp/dt). $V_{g}$ denotes the geostrophic wind vector ($V_{g}=\frac{1}{f_{0}}k\times \nabla \Phi$), $\zeta_{g}=\frac{1}{f_{0}}\nabla^{2}\Phi$ is the geostrophic relative vorticity, and *f* is the Coriolis parameter with $f_{0}$ as its reference value. *T* represents the air temperature (K), *R* is the gas constant for dry air, and $\sigma =-\frac{\alpha }{\theta }\frac{\partial \theta }{\partial p}$ denotes the static stability parameter, where α is the specific volume and θ is the potential temperature. The first term on the right-hand side represents the vertical derivative (differential) of geostrophic absolute vorticity advection, and the second term denotes the horizontal Laplacian of geostrophic thermal advection.

#### 3.3.5. Water Vapor Flux Convergence Feature Field ($F_{qflux}$)

The water vapor flux at 850 hPa is calculated as follows:

(5) Q=q·V

In Equation (5), *q* is the specific humidity (units: g/kg); and *V* is the horizontal wind vector (units: m/s). Further, the horizontal divergence of the water vapor flux is computed to quantify the convergence intensity of water vapor, as expressed in the following formula:

(6) $$-\nabla \cdot Q=-\left(\frac{\partial (qu)}{\partial x}+\frac{\partial (qv)}{\partial y}\right)$$

In Equation (6), u is the zonal wind component (units: m/s ); v is the meridional wind component (units: m/s); where *x* and *y* represent the spatial Cartesian coordinates mapped via a metric conformal projection with units of meters. Calculated strictly under this constant metric distance framework, the negative value of the water vapor flux divergence accurately quantifies physical water vapor convergence. By processing this field, the water vapor flux convergence intensity feature field $F_{qflux}$ is generated to indicate the key areas where water vapor accumulation is required for precipitation.

### 3.4. Construction of Feature Tensor

After calculating the aforementioned physical diagnostic fields, all the fields are strictly aligned in terms of time, space, and resolution. They are then stacked along the dimension of physical variables to form a unified multi-dimensional feature tensor. Specifically, the feature tensor for each sample is systematically formatted as a three-dimensional structure: [number of physical feature channels, longitude, latitude], wherein variables extracted from distinct isobaric levels and surface layers are directly concatenated along the feature channel dimension. This feature tensor serves as the standard input for the subsequent machine learning model, achieving the structured organization of multi-source meteorological information within a unified spatiotemporal framework. It provides a foundational data carrier for integrating dynamic diagnostics and statistical learning.

### 3.5. Model Design

#### 3.5.1. Input Data

The input data for this method consists of a set of predefined meteorological and geographic feature fields, totaling 18 data fields, which are divided into the following three categories:

**(a) Basic Numerical Forecast Fields.** This category includes 10 specific physical quantities, seamlessly integrating thermodynamic outputs from the 850 hPa isobaric level with targeted surface and column-integrated dynamic metrics. These fields are:

- Dynamic Fields: Horizontal wind field (U, V), vertical velocity field (OMEGA);
- Thermodynamic and Surface-Column Metrics: This incorporates the 850 hPa geopotential height (Z) and temperature (T) fields, alongside the column-integrated convective available potential energy (CAPE) and the two-dimensional surface pressure field (PSFC);
- Moisture Fields: Specific humidity field (Q), relative humidity field (RH), and precipitation forecast field (PRE).

**(b) Static Geographic Information Fields.** This category contains 3 features used to describe surface characteristics: High-resolution digital elevation model (DEM), slope, and aspect.
**(c) Diagnostic Feature Fields.** This category contains 5 intermediate fields generated through the physical diagnostic steps outlined above. These fields directly indicate precipitation potential: Low-level shear line feature field ($F_{shear}$), mid-to-upper-level trough-ridge impact feature field ($F_{tr}$), upper-level jet stream dynamic forcing feature field ($F_{jet}$), quasi-geostrophic omega equation vertical motion field ($F_{\omega }$), and water vapor flux convergence feature field ($F_{qflux}$).

#### 3.5.2. Encoder Architecture

The encoder is composed of four sequentially connected layers. Each layer consists of two consecutively connected residual convolution blocks, where each block contains two 3 × 3 convolution layers and an identity residual connection [29]. Except for the fourth layer, each layer is followed by a 2 × 2 max-pooling layer to downsample the feature maps spatially. The initial number of channels ($C_{1}$) in the encoder is set to 64, and the number of channels doubles with each subsequent layer, with the output channel numbers being 64, 128, 256, and 512 for each layer After three downsampling operations, the height and width of the output feature maps are reduced to 1/8 of the original input size, while the channel dimension increases to 512, thereby enabling multi-scale, deep-level abstraction of the input features.

#### 3.5.3. Attention Mechanism

After the bottleneck layer (i.e., the output of the final encoder layer), an adaptive spatial attention module is added to generate a spatial weight map that focuses on the key regions for precipitation prediction. First, channel attention is computed on the feature map output from the bottleneck layer (with 512 channels) by performing global average pooling and global max pooling. These produce two 512-dimensional channel description vectors. These vectors are summed and passed through a two-layer multi-layer perceptron (MLP), where the first layer reduces the dimension from 512 to 64, and the second layer restores it from 64 to 512. A Sigmoid activation function is applied to generate the channel attention weight vector. This vector is then multiplied element-wise with the original bottleneck feature map, re-weighting the features in the channel dimension [30].

Next, spatial attention is applied to the channel-weighted feature map. Along the channel dimension, both the mean and maximum values are computed to generate two single-channel feature maps (with spatial dimensions of (H/8×W/8)). These maps are concatenated along the channel dimension, passed through a 7 × 7 convolution layer for further fusion, and output as a single-channel feature map. A Sigmoid activation function is then applied to generate the spatial attention weight map. Finally, this weight map is multiplied element-wise with the channel-weighted feature map, resulting in a final feature representation modulated by both channel and spatial attention. This attention module is the main component explicitly derived from the bio-inspired selective-attention principle: instead of treating all grid points and feature channels equally, the network allocates greater representational weight to dynamically salient regions and variables.

#### 3.5.4. Decoder Architecture

The decoder is used to reconstruct the high-resolution precipitation forecast field, employing a single-branch U-Net-based decoder structure. The decoder consists of three layers, each progressively restoring the spatial dimensions of the feature maps and integrating feature information from different stages of the encoder [31]. The design is as follows:

Each decoding layer performs the following operations in sequence: First, bilinear interpolation is used for 2× upsampling, doubling the size of the feature maps. Then, the upsampled feature maps are concatenated with the corresponding encoder layer feature maps to achieve cross-scale feature fusion. The fused feature maps are then passed through two consecutive residual convolution blocks, each containing two 3 × 3 convolution layers and corresponding residual connections, as in the encoder.

The number of channels in the decoder decreases progressively from 512 channels in the bottleneck layer, with the output channel numbers being 256, 128, 64, and 32. After three layers of feature restoration and fusion, the final layer applies a 1 × 1 convolution to map the 32-channel feature maps to a single-channel output. The Softplus activation function is used to constrain the output values, ensuring that the precipitation forecast results are non-negative and consistent with the physical nature of precipitation.

### 3.6. Training-Time Regulatory Physical Constraint Loss Function

The physical constraint loss function is designed as a training-time regulatory component in the proposed bio-inspired framework. The constraint terms guide the neural network to produce precipitation fields that are not only numerically close to observations but also consistent with water-vapor supply and terrain-induced lifting mechanisms. This mechanism should be understood as constraint-based regulation during optimization, not as a recurrent sensing–comparator–effector feedback loop during inference. The loss function consists of three weighted components: data reconstruction loss, water vapor conservation constraint loss, and terrain forcing constraint loss [32]. The total loss function *L* is defined as:

(7) $$L=L_{data}+\lambda_{1}L_{mass}+\lambda_{2}L_{terrain}$$

In Equation (7), $L_{data}$ is the data reconstruction loss term, which measures the direct deviation between the predicted and true values; $L_{mass}$ is the water vapor conservation constraint loss term, which couples precipitation with the physical relationship of water vapor convergence; $L_{terrain}$ is the terrain forcing constraint loss term, which incorporates the dynamic influence of terrain on precipitation; $\lambda_{1}$ and $\lambda_{2}$ are non-negative hyperparameters that adjust the relative weight of the two physical constraints in the total loss. During the specific training implementation, the initial values of $\lambda_{1}$ and $\lambda_{2}$ are both set to 0.1 to ensure that the data reconstruction loss dominates the early training stage, while the constraint terms gradually exert regulatory effects.

The data reconstruction loss is measured using Mean Squared Error (MSE) to evaluate the difference between the predicted precipitation field and the true precipitation field, as shown in Equation (8):

(8) $$L_{data}=\frac{1}{N}\sum_{i=1}^{N}{\left(P_{pred}^{\left(i\right)}-P_{true}^{\left(i\right)}\right)}^{2}$$

In Equation (8), *N* is the total number of valid grid points in a training batch; $P_{pred}^{\left(i\right)}$ is the predicted precipitation value at the *i*-th spatial grid point; and $P_{true}^{\left(i\right)}$ is the corresponding true observed precipitation value.

A simplified moisture convergence penalty is introduced as a computationally efficient grid-level dynamic proxy. Recognizing the inherent descriptive defects caused by the single-layer approximation and the resulting omission of full three-dimensional vertical integration, this penalty encourages the model to correlate precipitation areas with the diagnostic 850 hPa water vapor flux convergence. To compensate for the incomplete representation of complex topographic precipitation mechanisms, adaptive scaling parameters α and β are introduced. Sensitivity analyses conducted during the validation phase indicate that setting the constraint weights $\lambda_{1}$ and $\lambda_{2}$ to 0.1 allows the model to flexibly adjust α to simulate column-integrated moisture effects, effectively mitigating the structural errors associated with the two-dimensional simplifications. This is expressed in Equation (9):

(9) $$L_{mass}=\frac{1}{N}\sum_{i=1}^{N}{\left[P_{pred}^{\left(i\right)}-\max\left(0,-\alpha \cdot Q_{diag}^{\left(i\right)}\right)\right]}^{2}$$

In Equation (9), $Q_{diag}^{\left(i\right)}$ represents the diagnostic water vapor flux divergence calculated from the input meteorological fields at the *i*-th spatial grid point; α is a learnable scaling parameter, initially set to 1.0, and iterated during each model training with a range of [0–3]. Its role is to adaptively adjust the scaling relationship between water vapor flux divergence and precipitation rate, compensating for biases caused by single-layer approximations and unit conversions; the operation $\max(0,-\alpha \cdot Q_{diag}^{\left(i\right)})$ ensures that the physical constraint applies only to the water vapor convergence region and not to the water vapor divergence region, consistent with the physical understanding that precipitation originates from water vapor convergence.

A topographic modulation term is formulated to penalize instances where heavy precipitation fields starkly contradict the underlying terrain uplift zones. Instead of forcing absolute geometric alignment, this soft constraint encourages the network to recognize the generalized precipitation enhancement effect of windward slopes under favorable moisture and atmospheric stability conditions, and the intensity of this change should be proportional to the terrain slope and the degree of windwardness. This guides the model to learn the classic physical mechanism of terrain-induced precipitation, such that precipitation increases with altitude on windward slopes. The terrain forcing constraint loss is expressed in Equation (10):

(10) $$L_{terrain}=\frac{1}{N}\sum_{i=1}^{N}{\left[\nabla P_{pred}^{\left(i\right)}\cdot n_{terrain}^{\left(i\right)}-\beta \cdot S^{\left(i\right)}\cdot \cos\left(\varphi^{\left(i\right)}-\theta \right)\right]}^{2}$$

In Equation (10), $\nabla P_{pred}^{\left(i\right)}$ is the spatial gradient vector of the predicted precipitation field at the *i*-th grid point, indicating the direction of maximum precipitation increase, and its magnitude represents the rate of change; $n_{terrain}^{\left(i\right)}$ is the unit vector in the terrain gradient direction at the *i*-th grid point, pointing toward the steepest uphill direction; $S^{\left(i\right)}$ is the slope value at the *i*-th grid point, derived from the input slope field, representing the steepness of the terrain; $\varphi^{\left(i\right)}$ is the aspect angle at the *i*-th grid point, provided by the input aspect field; β is a learnable intensity coefficient, initially set to 0.05, iterated during each model training with a range of [0–0.5]; and $\cos\left(\varphi^{\left(i\right)}-\theta \right)$ measures the relative relationship between slope aspect and prevailing wind direction. When the slope aspect faces the prevailing wind, the cosine value approaches 1, indicating the strongest terrain uplift effect; when the slope is lee-facing, the cosine value is negative, indicating a reduced or even suppressed precipitation effect.

### 3.7. Training Strategy

A progressive training strategy is adopted for model training:

- Warm-up Phase: During this phase, only the data loss is used to train the model for 10 epochs, allowing the model to initially learn the spatial distribution of precipitation.
- Main Training Phase: In this phase, the physical constraint loss is introduced, and the model is trained for 50 epochs to allow it to incorporate physical laws along with data learning.
- Fine-tuning Phase: The learning rate is reduced, and training continues for another 50 epochs to fine-tune the model and improve its performance.

This staged approach helps gradually integrate physical constraints while ensuring that the model maintains a solid understanding of the underlying data structure [33].

The specific process of the precipitation correction method proposed in this paper is shown in Algorithm 1.
**Algorithm 1** Physically Constrained Precipitation Correction (PCPNet)Input: NWP forecast fields F, geographic data G, pre-trained model parameters θ Output: Corrected precipitation field P 1: Normalize F and G to 0.01° resolution, crop to target region → F′, G′ 2: Compute diagnostic feature fields from F′: (a) $F_{shear}$ ← shear_vorticity (850 hPa U, V) (b) $F_{tr}$ ← trough_ridge_index (500 hPa Z) (c) $F_{jet}$ ← jet_dynamic_forcing (200 hPa U, V, T) (d) $F_{omega}$ ← vertical_motion (500 hPa ζ, D) (e) $F_{qflux}$ ← moisture_convergence (850 hPa Q, U, V) 3: Stack F′, G′, $F_{shear}$, $F_{tr}$, $F_{jet}$, $F_{omega}$, $F_{qflux}$ into tensor X 4: Load encoder–attention–decoder model M with parameters θ 5: P ← M(X) 6: Return P --------- Training Summary (for reference) --------- During training, loss = MSE(P, O) + $\lambda_{1}$·MSE(P, $\max\left(0,-\alpha \cdot F_{qflux}\right)$) + $\lambda_{2}$·MSE(∇P·$n_{terrain}$, β·slope·cos(aspect θ)) with learnable α, β and parameters θ updated via gradient descent.


## 4. Dataset

### 4.1. Observation Data and Live Data

The observation data consists of hourly data from 98 meteorological observation stations in Hunan Province from 2021 to 2023, which are used as the true values in this study. The latitude and longitude of these stations range from 24° N–31° N and 107° E–115° E. The data is interpolated using bilinear interpolation, and K-fold cross-validation experiments are performed. As a result, approximately 200,000 valid site samples are obtained. The hourly precipitation data is categorized into four levels, as shown in Table 1. The data distribution is highly imbalanced, with the “no precipitation” category accounting for more than 88% of the total data, while moderate and higher precipitation events account for only 0.1%.

The live data is sourced from Hunan Province, with a temporal resolution of one hour and a time range from 2021 to 2023. The grid resolution is 0.01°× 0.01°. This real-time data serves as the target for correction in our study.

### 4.2. Data Preprocessing Methods

The following steps were taken in the data preprocessing:

(1) Outlier and Missing Value Handling: Due to the influence of observational environment, errors, and algorithms, the dataset contained outliers and missing values. These were removed through data cleaning to ensure data quality.
(2) Data Assimilation: For datasets with inconsistent resolution and grid standards, interpolation and other methods were used to assimilate the data onto a unified grid with consistent resolution.

### 4.3. Dataset Partitioning

In this study, a temporal block-based K-fold cross-validation method was employed to alleviate instability caused by the randomness of training data and to rigorously prevent temporal data leakage. The entire dataset spans from January 2021 to December 2023 over the complete Hunan Province spatial grid. Specifically, the sample data collected from 2021 to 2022 was sequentially divided into five strictly non-overlapping temporal subsets, ensuring that the proportion of precipitation in each subset was consistent with the overall sample distribution [34]. During the cross-validation phase, four subsets were used for training and one for internal validation. The completely independent data from the entire year of 2023 was reserved exclusively for final model testing. Furthermore, all features and targets were uniformly aligned on the spatial grid, ensuring that spatial autocorrelation among adjacent observation stations did not induce spatial information leakage during the evaluation phase. During the model optimization phase, four subsets were used as training data, while the remaining subset served as the internal validation set to monitor convergence and prevent overfitting. This approach allowed the model to be trained and tested on each subset, with the average of the five experimental results used as an indicator for evaluating the model’s performance. This method enhanced the model’s generalization ability and significantly improved its stability and accuracy when handling different data distributions.

## 5. Experiments and Results

The experiments were conducted using the PyTorch 2.4 deep learning framework and Python 3.10 as the development language. The hardware configuration included an Intel Core i9-9700F processor and an NVIDIA RTX A5000 graphics card. During the training process, the Adam optimizer was selected, with $\beta_{1}$ and $\beta_{2}$ representing the weight coefficients for the moving average of the gradient and the moving average of the squared gradient, respectively. $\beta_{1}$ and $\beta_{2}$ were set to 0.5 and 0.999, respectively. The batch size was 256, the initial learning rate was set to 0.001, and the training was conducted over 110 epochs.

### 5.1. Evaluation Metrics

To evaluate the final correction performance and generalization capability of the proposed model, all reported statistical metrics and comparative evaluations are strictly derived from the independent 2023 out-of-sample test dataset. These independent predictions were then compared with the uncorrected CMADAAS real-time data. To ensure absolute fairness in comparative evaluations, all benchmark models including Linear Regression [35], Bagging, Boosting [36], MLP, TabNet [37], and TPM [38] were fed the identical augmented multi-dimensional feature tensor used by PCPNet. This uniform input space comprehensively encompasses the basic numerical forecast fields, static geographic information fields, and the five explicitly extracted weather-system diagnostic feature fields. Furthermore, hyperparameter optimization for all baseline models was rigorously conducted using grid search over the cross-validation sets, employing the identical early stopping training strategy to achieve their respective optimal convergence states. The ablation model that does not consider weather systems is referred to as PhysNet, while the complete architecture is referred to as PCPNet.

To comprehensively assess the data consistency before and after correction, five common evaluation metrics were selected: Mean Absolute Error (MAE), Mean Squared Error (MSE), Mean Relative Error (MRE), Mean Absolute Relative Error (MARE), and Correlation Coefficient (CC), as shown in Equations (11) through (15).

(11) $$MAE=\frac{1}{n}\sum_{i=1}^{n}\left|P_{i}-G_{i}\right|$$

(12) $$MSE=\frac{1}{n}\sum_{i=1}^{n}{\left(P_{i}-G_{i}\right)}^{2}$$

(13) $$MRE=\frac{1}{n}\sum_{i=1}^{n}\frac{P_{i}-G_{i}}{G_{i}}$$

(14) $$MARE=\frac{1}{n}\sum_{i=1}^{n}\frac{\left|P_{i}-G_{i}\right|}{G_{i}}\times 100\%$$

(15) $$CC=\frac{\sum_{i=1}^{n}\left(G_{i}-\bar{G}\right)\left(P_{i}-\bar{P}\right)}{\sqrt{\sum_{i=1}^{n}{\left(G_{i}-\bar{G}\right)}^{2}}\cdot \sqrt{\sum_{i=1}^{n}{\left(P_{i}-\bar{P}\right)}^{2}}}$$

where: *n* is the number of samples, $P_{i}$ and $G_{i}$ represent the predicted and true values, respectively.

### 5.2. Experimental Results

**Figure 2:**
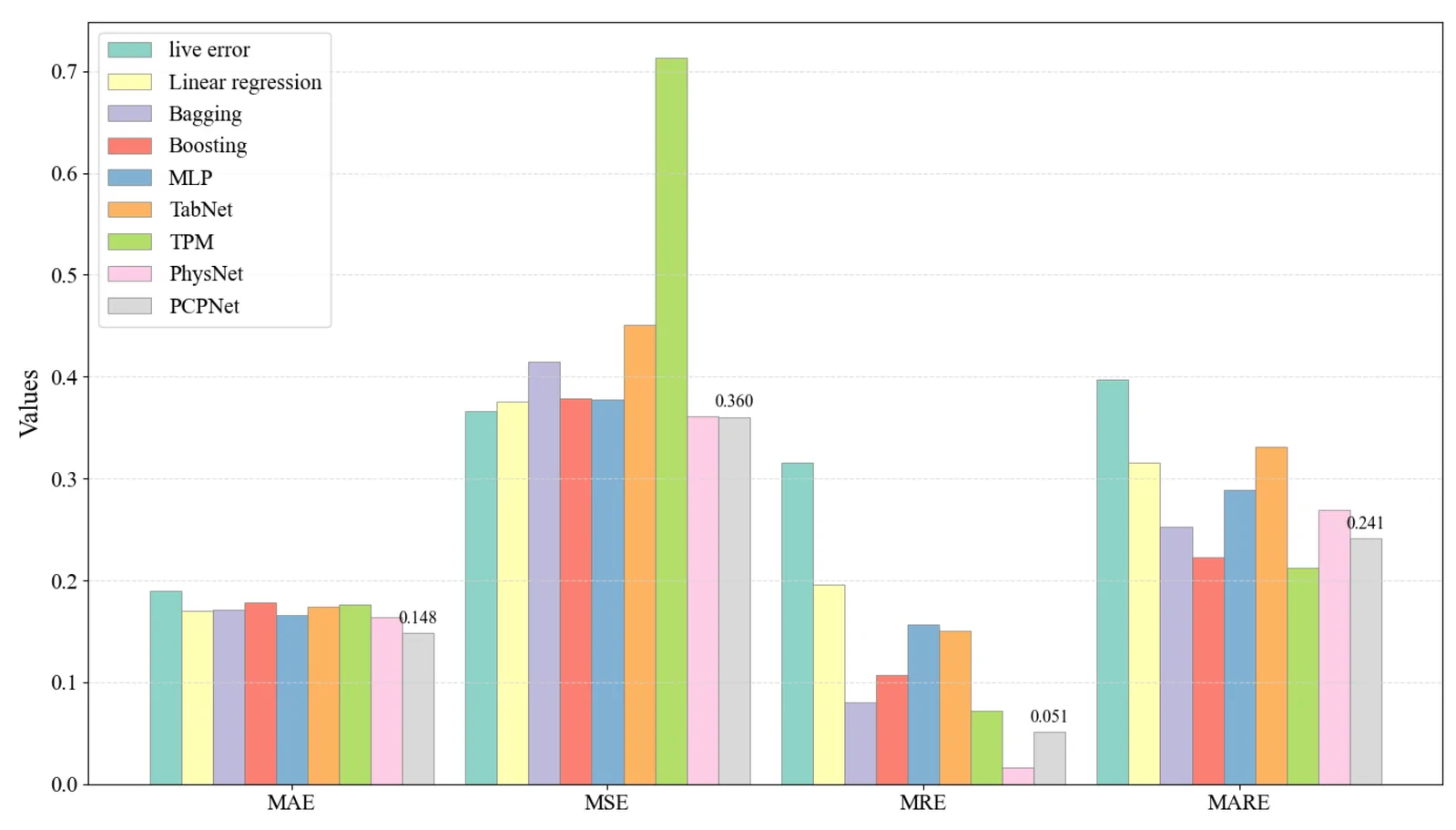
Comparison of Different Model Performance Metrics.

In the experimental results, the method proposed in this paper demonstrates excellent performance across multiple evaluation metrics, as shown in Figure 2 and Table 2.

To verify the statistical significance of the performance improvements, paired *t*-tests were conducted comparing the absolute errors of PCPNet against the benchmark models. The reductions in MAE and MSE achieved by PCPNet are statistically significant at the 95% confidence level with a p-value of less than 0.05, indicating that the incorporation of weather systems and physical constraints provides robust and non-random performance enhancements. Compared to other traditional models, the proposed method demonstrates significant advantages in both MAE and MSE. The MAE of the PCPNet method is 0.148, which is notably better than other methods such as Boosting (0.178) and MLP (0.166), indicating that incorporating weather system features can effectively enhance the model’s accuracy. Additionally, in terms of MSE, the proposed method achieves the best result with a value of 0.36, outperforming benchmark models such as TPM (0.713).

The MRE and MARE metrics effectively quantify the relative deviation magnitude normalized by the true precipitation intensity. PCPNet achieves an optimal MRE of 0.051 and a MARE of 0.241, indicating its superior capability to tightly constrain the relative amplitude of overestimation or underestimation during intense precipitation fluctuations. Regarding spatial pattern consistency, the Correlation Coefficient metric confirms that PCPNet reaches 0.979, indicating that the corrected grid preserves the morphological evolution of the weather systems.

### 5.3. Special Case Study

To further validate the model’s performance, we analyzed the observed precipitation data from 00:00 to 23:00 on 9 May 2023, and its corresponding correction results. Precipitation data at four time points—00:00, 06:00, 12:00, and 18:00—was corrected using different methods. By conducting a quantitative evaluation of the precipitation correction results from different methods, we observed that the MLP model showed the smallest improvement, likely because MLP only fits the relationships between features in a nonlinear manner.

To assess the accuracy of the methods, we calculated the Mean Absolute Error (MAE) between the observed values, corrected values, and the observations at station sites. Table 3 summarizes the MAE values for precipitation corrections using Linear Regression, Bagging, Boosting, MLP, TabNet, TPM, PhysNet, and PCPNet methods at 00:00, 06:00, 12:00, and 18:00 on 9 May 2023. The results show that at 00:00 and 06:00, the PCPNet method performed the best, with MAE values of 0.625 and 0.610, respectively. At 12:00, the Boosting method showed slightly better performance. However, at 18:00, PCPNet again outperformed the other methods with an MAE of 1.127.

Table 4 provides a detailed comparison of the real-time errors at observation station sites and the corrected errors using PCPNet for each hour from 00:00 to 23:00. The results show that PCPNet effectively reduced prediction errors at most time points, especially during periods of higher precipitation, demonstrating higher accuracy and stability.

**Figure 3:**
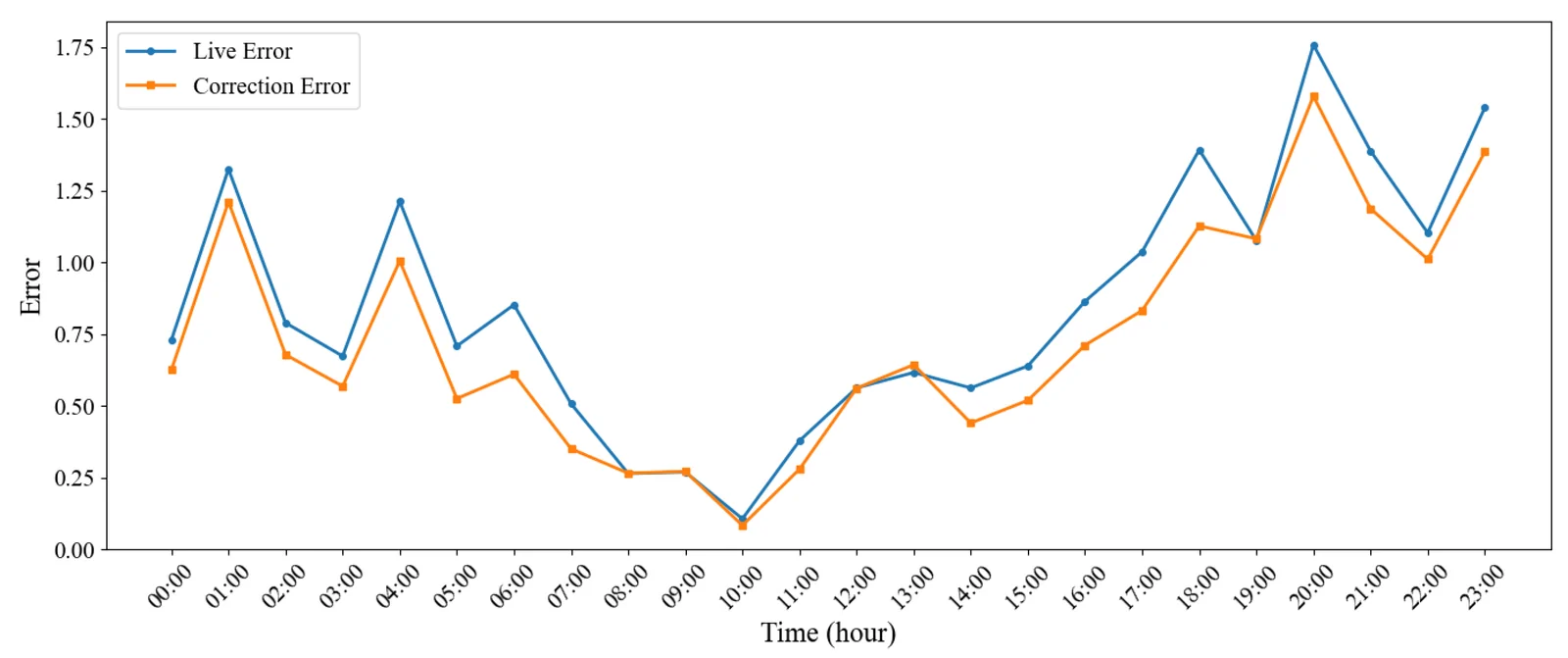
Comparison of live error and corrected error on 9 May 2023. The lines connect discrete hourly observation points to illustrate the temporal evolution trend.

The correction results were further visualized to demonstrate the superiority of the proposed correction method. As shown in Figure 3, the error is the absolute difference from the actual observed values, reflecting how close the predictions are to the observations. Figure 3 visually indicates that the correction errors from PCPNet represent a quantitative reduction compared to the real-time errors. The average value of the real-time errors is 0.848, while the average value of the corrected errors is 0.731, which is 13.77% lower than the real-time errors. Although there are four data points, specifically 08:00, 09:00, 13:00, and 19:00, where the uncorrected real-time error is smaller than the corrected error, the differences remain within a narrow margin ranging from 0.001 mm to 0.027 mm, which has virtually no impact on the precipitation analysis.

**Figure 4:**
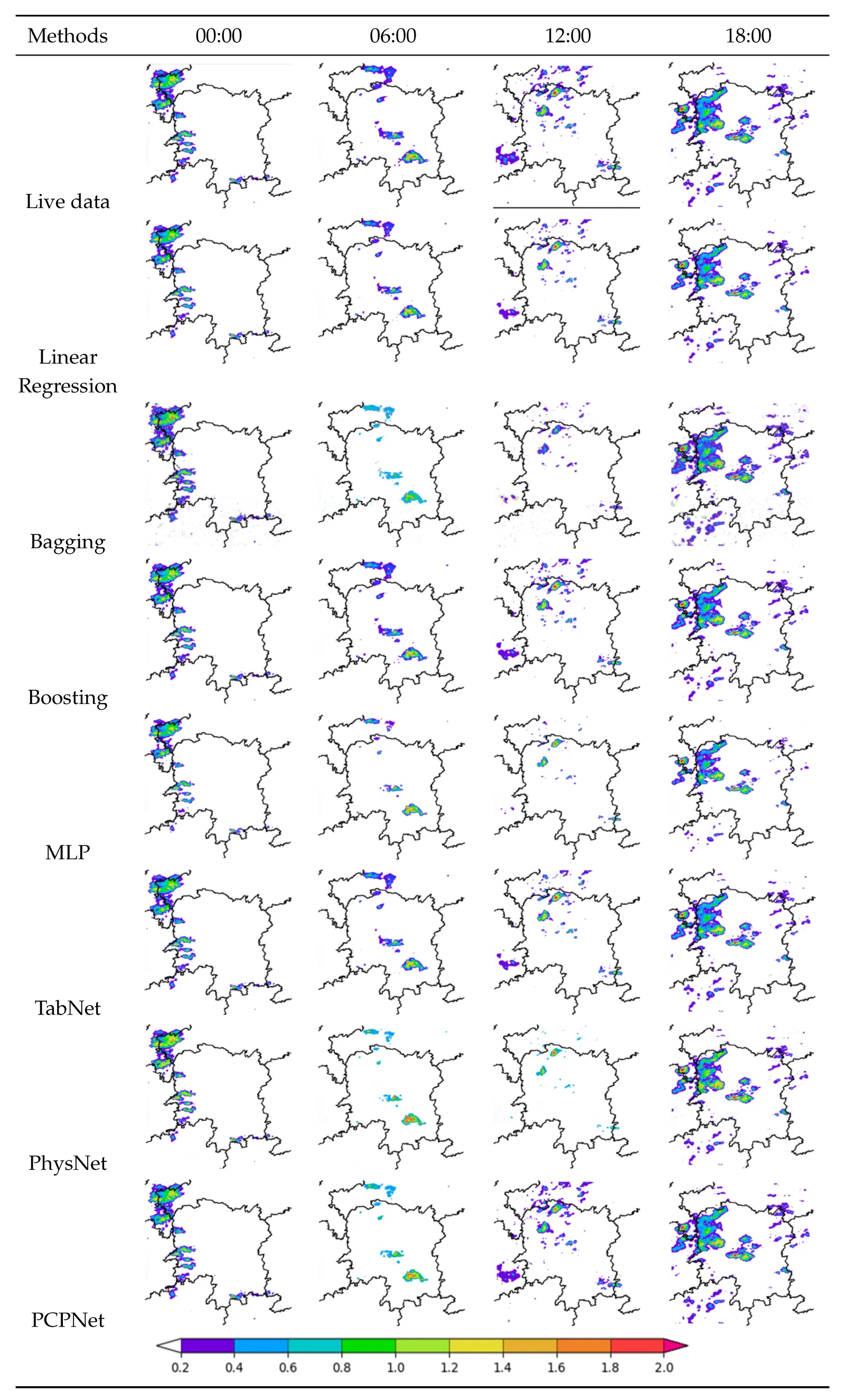
Visual comparison of correction results from different methods on 9 May 2023. Pseudocolors represent precipitation rates in millimeters per hour.

As shown in Figure 4, a visual comparison is made between the observed precipitation data and the results from seven correction methods at different time points (00:00, 06:00, 12:00, and 18:00) on 9 May 2023. The figure uses pseudocolors to represent precipitation, clearly displaying differences in the spatial distribution and intensity of precipitation.

It can be observed that linear regression captures the precipitation areas well at certain time points, but there are still biases in correcting the precipitation intensity. The Bagging method is closer to the spatial distribution of the observed data, though it overestimates or underestimates precipitation intensity in some regions. The Boosting method better reflects the intensity and distribution of observed precipitation data across multiple time points, though some errors remain in certain periods. The MLP method performs well in capturing precipitation distribution, similar to the Bagging method, but tends to overestimate precipitation in higher-precipitation areas.

To robustly assess model stability under extreme class imbalance where heavy precipitation events constitute only 0.02% of the dataset, bootstrap resampling with 1000 iterations was conducted to establish 95% confidence intervals for performance metrics across graded precipitation thresholds. From Figure 4 and the resampled statistical distributions, it is evident that the proposed PCPNet method prioritizes capturing extreme weather signals. While it does not pursue absolute precision superiority across every minor precipitation fluctuation, PCPNet exhibits a significantly high recall rate for moderate-to-heavy precipitation events. By leveraging jet-stream dynamic forcing features, the model successfully restores the core intensity centers of heavy precipitation systems, drastically reducing the missed detection rate for extreme rainfall compared to purely statistical baselines. Methods such as MLP and Bagging exhibit widespread over-smoothing, generating large areas of unrealistic light precipitation and failing to accurately capture the central core intensity of storm systems. Conversely, guided by the water vapor flux convergence and low-level shear diagnostic fields, PCPNet effectively suppresses false precipitation triggers in thermodynamically stable regions. Furthermore, the terrain forcing constraint enables PCPNet to render a more physically consistent spatial structure over windward and leeward slopes. Particularly in areas with higher precipitation, the correction performance of PCPNet is clearly superior to the other methods.

### 5.4. Precipitation Correction Enhancement via Weather Systems Analysis

To compare the effectiveness and generalization capabilities of various models, we selected the top four models based on the MAE metric: MLP, Linear Regression, PhysNet, and the proposed PCPNet model. These models were tested using the 2023 dataset.

**Figure 5:**
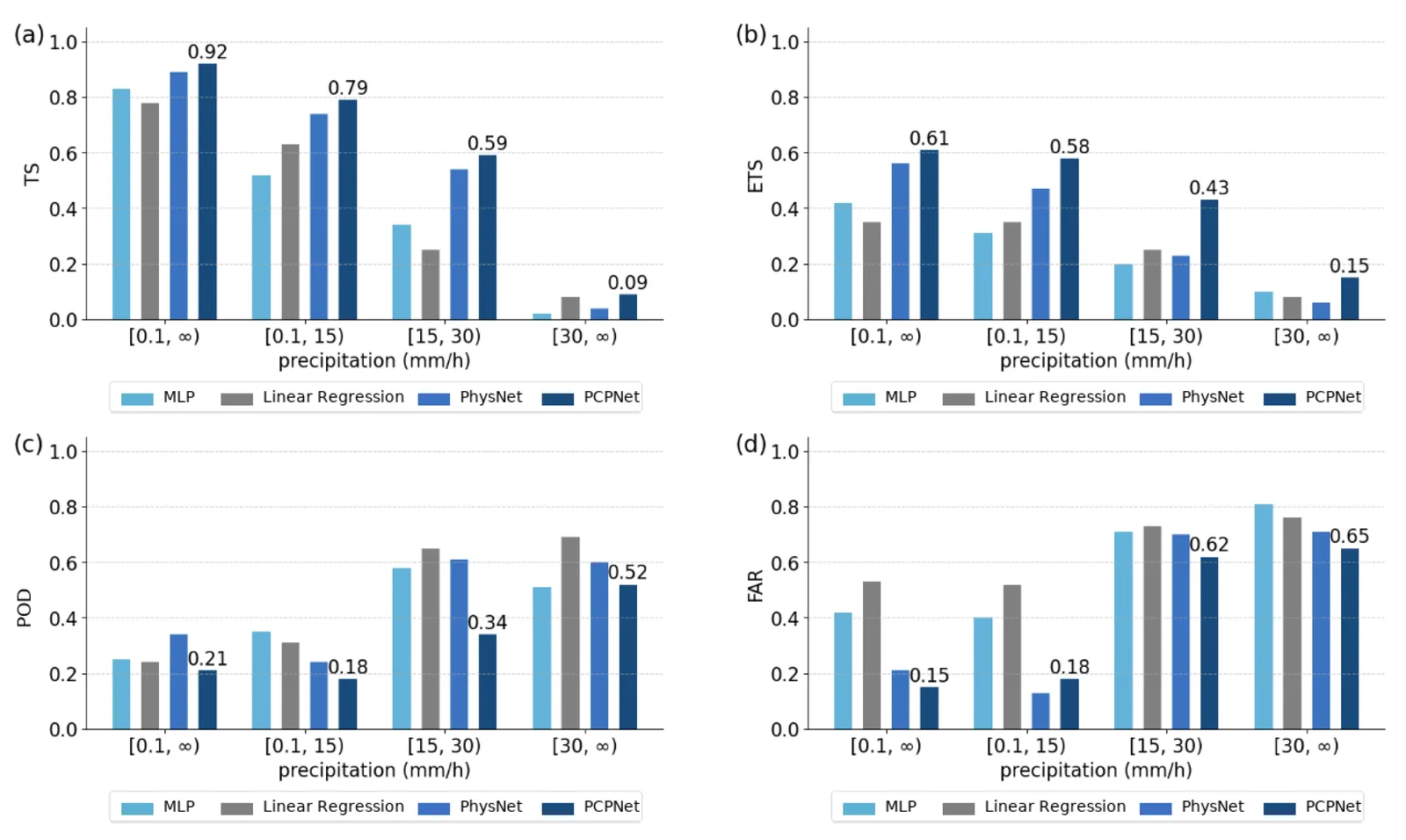
Evaluation metrics of different correction models on the 2023 dataset: (**a**) TS score, (**b**) ETS score, (**c**) Probability of Detection, and (**d**) False Alarm Ratio.

As shown in Figure 5a, the proposed correction model outperforms the other models in terms of TS (Threat Score). PCPNet and PhysNet perform better than MLP and Linear Regression in the weak precipitation range, indicating that the proposed model excels in handling weak precipitation. PCPNet and PhysNet maintain a significant advantage in the moderate precipitation range, especially in high-precipitation areas, where traditional models like MLP and Linear Regression are nearly ineffective. Although all models have relatively lower TS scores in the heavy precipitation range, PCPNet still achieves the highest value among the four models. This suggests that the proposed model provides a consistent advantage across precipitation thresholds, although heavy precipitation remains the most difficult category.

The difference between PhysNet and the proposed model highlights the crucial role of incorporating weather systems into precipitation correction. Specifically, in the weak-to-moderate precipitation range, integrating weather system feature fields significantly improved the performance of the PCPNet model, indicating that the model enhanced its ability to capture meteorological variations in these ranges.

Additionally, the Probability of Detection (POD) comparison in Figure 5c and the False Alarm Ratio (FAR) comparison in Figure 5d show how the weather-system features change event detection behavior. For light precipitation, PCPNet has a lower POD than PhysNet, indicating a more conservative detection tendency. However, in the moderate and heavy precipitation ranges, PCPNet maintains competitive POD values while reducing FAR relative to PhysNet. This pattern indicates that incorporating weather-system feature fields, especially jet-stream-related forcing, helps suppress false alarms and improves the reliability of moderate-to-heavy precipitation detection.

## 6. Conclusions

This paper proposes a grid-based precipitation correction method that integrates weather system diagnostics with physical constraints, aiming to address the dual challenges of the lack of physical interpretability in statistical methods and the inefficiency of manual diagnostics in existing technologies. The core of this method is the construction of a diagnosis–constraint–correction workflow rather than an inference-time closed feedback loop. First, key weather system features such as shear lines, jet stream forcing, and water vapor convergence are quantified, transforming the traditional subjective experience of forecasters into learnable physical diagnostic fields. These features are then fused with the original meteorological data and input into a deep learning model based on an encoder–attention–decoder architecture. More importantly, during the model training process, two differentiable physical constraint loss functions—water vapor conservation and terrain forcing—are introduced to encourage the model output to follow fundamental physical laws while optimizing for numerical accuracy.

The experimental results show that this method outperforms traditional approaches in statistical metrics such as MAE and MSE, while also demonstrating stronger physical consistency and forecasting stability in complex weather processes. Overall, the proposed method represents a step from purely statistical fitting toward bio-inspired physical intelligence, in which environmental sensing, selective information processing, and regulatory physical constraints are jointly used to improve precipitation correction. 

## Figures and Tables

**Table 1:** Classification Table of Precipitation.

Precipitation Level	Precipitation Intensity (mm)	Sample Count	Sample Proportion (%)
No Precipitation	[0, 0.1)	1,581,703	88.45
Light Precipitation	[0.1, 15)	204,812	11.45
Moderate Precipitation	[15, 30)	1577	0.08
Heavy Precipitation	[30, ∞)	334	0.02

**Table 2:** Comparison of experimental results of various methods on the test set. Bold values indicate the best result for each metric.

Evaluation Metrics	Live Error	Linear Regression	Bagging	Boosting	MLP	TabNet	TPM	PhysNet	PCPNet
MAE	0.190	0.170	0.171	0.178	0.166	0.174	0.176	0.164	**0.148**
MSE	0.366	0.376	0.415	0.379	0.378	0.451	0.713	0.361	**0.360**
MRE	0.316	0.196	0.080	0.107	0.157	0.15	0.072	**0.016**	0.051
MARE	0.397	0.316	0.253	0.223	0.289	0.331	**0.212**	0.269	0.241
CC	0.979	0.979	0.977	0.978	0.979	0.978	0.714	0.978	**0.979**

**Table 3:** Comparison of MAE for different precipitation correction methods on 9 May 2023. Bold values indicate the lowest MAE at each time.

Time	Live Error	Linear Regression	Bagging	Boosting	MLP	TabNet	TPM	PhysNet	PCPNet
00:00	0.729	0.757	0.727	0.709	0.774	0.756	0.728	0.700	**0.625**
06:00	0.852	0.884	0.817	0.859	0.948	0.883	0.852	0.812	**0.610**
12:00	0.562	0.550	0.566	**0.524**	0.556	0.574	0.562	0.565	0.561
18:00	1.392	1.412	1.422	1.375	1.489	1.429	1.391	1.367	**1.127**

**Table 4:** Comparison of corrected error on 9 May 2023. Bold values indicate the lower error between the live and corrected forecasts at the same hour.

Time	Live Error	Correction Error	Time	Live Error	Correction Error
00:00	0.729	**0.625**	12:00	**0.562**	0.561
01:00	1.325	**1.211**	13:00	**0.616**	0.643
02:00	0.789	**0.678**	14:00	0.562	**0.440**
03:00	0.673	**0.568**	15:00	0.639	**0.519**
04:00	1.212	**1.005**	16:00	0.864	**0.711**
05:00	0.708	**0.525**	17:00	1.037	**0.832**
06:00	0.852	**0.610**	18:00	1.392	**1.127**
07:00	0.507	**0.350**	19:00	**1.076**	1.082
08:00	**0.264**	0.265	20:00	1.758	**1.579**
09:00	**0.269**	0.271	21:00	1.388	**1.187**
10:00	0.106	**0.083**	22:00	1.103	**1.011**
11:00	0.378	**0.279**	23:00	1.539	**1.385**

## Data Availability

The datasets used in this study are not publicly available because they contain operational meteorological products provided under institutional data-use restrictions. Data may be made available from the corresponding author upon reasonable request and with permission from the Hunan Meteorological Bureau.

## References

[1] Vincent J.F.V., Bogatyreva O.A., Bogatyrev N.R., Bowyer A., Pahl A.-K. (2006). Biomimetics: Its practice and theory. J. R. Soc. Interface.

[2] Itti L., Koch C., Niebur E. (1998). A model of saliency-based visual attention for rapid scene analysis. IEEE Trans. Pattern Anal. Mach. Intell..

[3] Glahn H.R., Lowry D.A. (1972). The use of model output statistics (MOS) in objective weather forecasting. J. Appl. Meteorol..

[4] Wilks D.S., Hamill T.M. (2007). Comparison of ensemble-MOS methods using GFS reforecasts. Mon. Weather Rev..

[5] Gneiting T., Raftery A.E., Westveld A.H., Goldman T. (2005). Calibrated probabilistic forecasting using ensemble model output statistics and minimum CRPS estimation. Mon. Weather Rev..

[6] Homleid M. (1995). Diurnal corrections of short-term surface temperature forecasts using the Kalman filter. Weather Forecast..

[7] Jiang H.R., Fang W. (2024). Survey of application of deep learning in meteorological data correction. J. Comput. Appl..

[8] Fang H.B., Wang S.S., Wang X.L., Tan J.H., Lu L.B. (2024). Gridded temperature forecast correction method based on machine learning. Meteorol. Mon..

[9] Huang Z.Q., Zhao T.T.G., Tian Y., Wu Y., Li B., Chen X. (2024). Calibration and verification of monthly precipitation forecasts from gfdl-spear global climate model. South-To-North Water Transf. Water Sci. Technol..

[10] Liu Y., Zhao L., Li Z., Wang S., Ma Y., Meng X. (2025). Application evaluation of a bias correction method in the correction of CMIP6 precipitation data for summer in Qinghai-Xizang Plateau. Plateau Meteorol..

[11] Mendez M., Maathuis B., Hein-Griggs D., Alvarado-Gamboa L.F. (2020). Performance evaluation of bias correction methods for climate change monthly precipitation projections over Costa Rica. Water.

[12] Li Z., Liu F., Yang W., Peng S., Zhou J. (2021). A survey of convolutional neural networks: Analysis, applications, and prospects. IEEE Trans. Neural Netw. Learn. Syst..

[13] Grossberg S. (2013). Recurrent neural networks. Scholarpedia.

[14] Liu W., Wang Z., Liu X., Zeng N., Liu Y., Alsaadi F.E. (2017). A survey of deep neural network architectures and their applications. Neurocomputing.

[15] Tan L., Liu Q., Xia J. (2024). Deep learning based correction of ECMWF forecast products with fusion of DEM and FY-4A data. Acta Meteorol. Sin..

[16] Jiang Z., Zhao T., Li X., Cai H., Duan K., Wang H., Liu Z. (2023). Study on the calibration of ECMWF daily precipitation forecast based on Bernoulli-Gamma-Gaussian model. Water Resour. Hydropower Eng..

[17] Huang H.M., Chen J.X., Yao Q.F., Zhao L., Wei W.X., Xie L.S. (2024). Spatiotemporal distribution characteristics of short-duration heavy rainfall in Meizhou and the influence of terrain. Guangdong Meteorol..

[18] Zhang Q., Li J., Lu H., Zou H., Li K., Liang Y., Yang B., Liu J. (2025). TH-MuSiC: A high-performance multi-scale NWP-LBM coupling framework with CPU–GPU architecture for high-fidelity real-time urban wind field simulation. Build. Environ..

[19] Yan Z., Yang J., Zhang H., Li W., Wang Y., Liu H., Yu L. (2025). Biophysical feedback from earlier leaf-out enhances nonerosive precipitation in China. Commun. Earth Environ..

[20] Popescu M.-C., Balas V.E., Perescu-Popescu L., Mastorakis N. (2009). Multilayer perceptron and neural networks. WSEAS Trans. Circuits Syst..

[21] LeCun Y., Bottou L., Bengio Y., Haffner P. (1998). Gradient-based learning applied to document recognition. Proc. IEEE.

[22] Nair V., Hinton G.E. Rectified linear units improve restricted Boltzmann machines. Proceedings of the 27th International Conference on Machine Learning (ICML).

[23] Zhou T., Chang X., Lu H., Ye X., Liu Y., Zheng X. (2022). Pooling operations in deep learning: From “invariable” to “variable”. BioMed Res. Int..

[24] Holton J.R., Hakim G.J. (2013). An Introduction to Dynamic Meteorology.

[25] Hou J., Gao T. (2022). A method of shear line detection in vector fields based on descriptive statistics of circular data. Multimed. Tools Appl..

[26] Teubler F., Riemer M. (2021). Potential-vorticity dynamics of troughs and ridges within Rossby wave packets during a 40-year reanalysis period. Weather Clim. Dyn..

[27] Uccellini L.W., Kocin P.J. (1987). The interaction of jet streak circulations during heavy snow events along the East Coast of the United States. Weather Forecast..

[28] Bluestein H.B. (1993). Synoptic-Dynamic Meteorology in Midlatitudes. Volume II: Observations and Theory of Weather Systems.

[29] He K., Zhang X., Ren S., Sun J. Deep residual learning for image recognition. Proceedings of the IEEE Conference on Computer Vision and Pattern Recognition (CVPR).

[30] Woo S., Park J., Lee J.-Y., Kweon I.S., Ferrari V., Hebert M., Sminchisescu C., Weiss Y. (2018). CBAM: Convolutional block attention module. Computer Vision–ECCV 2018.

[31] Ronneberger O., Fischer P., Brox T., Navab N., Hornegger J., Wells W.M., Frangi A.F. (2015). U-Net: Convolutional networks for biomedical image segmentation. Medical Image Computing and Computer-Assisted Intervention–MICCAI 2015.

[32] Beucler T., Pritchard M., Rasp S., Ott J., Baldi P., Gentine P. (2021). Enforcing analytic constraints in neural networks emulating physical systems. Phys. Rev. Lett..

[33] Bengio Y., Louradour J., Collobert R., Weston J. Curriculum learning. Proceedings of the 26th International Conference on Machine Learning (ICML).

[34] Kohavi R. A study of cross-validation and bootstrap for accuracy estimation and model selection. Proceedings of the Fourteenth International Joint Conference on Artificial Intelligence (IJCAI).

[35] Montgomery D.C., Peck E.A., Vining G.G. (2021). Introduction to Linear Regression Analysis.

[36] Quinlan J.R. (1996). Bagging, boosting, and C4.5. Proc. Natl. Conf. Artif. Intell..

[37] Arik S.Ö., Pfister T. (2021). TabNet: Attentive interpretable tabular learning. Proc. AAAI Conf. Artif. Intell..

[38] Lin X., Chen X., Song L., Liu J., Li B., Jiang P. Tree based progressive regression model for watch-time prediction in short-video recommendation. Proceedings of the 29th ACM SIGKDD Conference on Knowledge Discovery and Data Mining.

